# “Like Walking into an Empty Room”: Effects of Eurythmy Therapy on Stress Perception in Comparison with a Sports Intervention from the Subjects' Perspective—A Qualitative Study

**DOI:** 10.1155/2015/856107

**Published:** 2015-03-10

**Authors:** B. Berger, M. Bertram, J. Kanitz, K. Pretzer, G. Seifert

**Affiliations:** ^1^Department of Health, University of Witten/Herdecke, 58313 Herdecke, Germany; ^2^Department of Nursing Science, University of Witten/Herdecke, 58313 Herdecke, Germany; ^3^Task Force Integrative Medicine, Department of Paediatrics Specialising in Oncology and Haematology, Charité University Hospital, 13353 Berlin, Germany

## Abstract

*Background*. Stress and health-related quality of life are important constructs used in treatment evaluation today. This study is based on a randomised controlled trial examining the stress-reducing effect of eurythmy therapy in comparison with step aerobics in 106 healthy but stressed subjects. The aim of the analysis was to characterise changes in the subjective perceptions of the participants. *Methods*. Interviews were conducted with 76 healthy adults, 36 (f = 31/m = 5) from the eurythmy group and 40 (f = 28/m = 12) from the step aerobics group both analysed by content analysis and phenomenologically. *Results*. The following categories were identified for the eurythmy therapy group:* enabling a productive therapeutic response, emergence of a new perceptual space, reevaluation of the accustomed perception, and emergence of new options for action*. Step aerobics places increased physical and intellectual demands. These are perceived differently as* pleasant and relaxing, insufficiently challenging and/or boring, and too challenging and thus experienced as stress-enhancing. Conclusion*. The qualitative results provided revealing insights into the profound effects of and subjective assignments of meaning to external and internal stress factors. Processes of mental reinterpretation leading to stress reduction can be stimulated by physical procedures such as eurythmy therapy.

## 1. Background

Stress and health-related quality of life are important constructs of treatment evaluation today. Stress reduction is considered a preventive health-relevant parameter. Programmes for lasting stress reduction prevent burnout and depressive episodes and can thus lead to reduction of various chronic somatic illnesses such as cardiovascular diseases and cancer.

In recent years, the two methods mindfulness based stress reduction (MBSR) and mindfulness based cognitive therapy (MBCT), in particular, have been increasingly employed as therapeutic techniques and also increasingly examined for their stress-reducing effects. The systematic review of randomised controlled studies conducted by Fjorback et al. in 2011 comes to the conclusion that MBSR can increase mental health and reduce stress symptoms as well as symptoms of anxiety and depression and can therefore be recommended as intervention for medical disease management [1].

Eurythmy therapy (EYT) is a treatment method used in anthroposophical medicine in which patients are taught exercises which integrate cognitive, emotional and volitional elements. The exercises are based on speech and direct the patient's attention to their own mentally experienced intentionality when performing the exercises. As a result, a connection between internal and external activity can be experienced.

With regard to the therapeutic effects of eurythmy therapy, emphasis has so far been placed on examining the interdependences between eurythmy therapy and physiological processes [2, 3]. An important result here was the increase in heart rate variability as an expression of the physiological reaction capacity of an organism, as well as the improvement of a circadian rhythm. With the exception of a few small pilot studies, for example, in children with brain tumours [4] or patients with hypertension, there are no clinical studies on eurythmy therapy. There is only a largish cohort study examining the long-term impact of EYT on disease score and quality of life in stressed but healthy subjects. In this study conducted by Kanitz and colleagues in 2011 the authors were able to show the effectiveness of eurythmy therapy with regard to improvement of stress coping and quality of life in healthy subjects [5].

In view of this dearth of studies, we decided to conduct a randomised controlled study to examine the effects of eurythmy therapy on stress coping and heart rate variability in healthy subjects. This prospective randomised three-arm study was conducted in 2010-2011 at the Berlin Charité hospital with 121 healthy subjects (publication in preparation). The transcripts of the interviews from this study form the data base for the present paper. The focus of the qualitative analysis was the subjective perception of the effectiveness of eurythmy therapy and step aerobics on the stress perception of the study participants.

## 2. Material and Methods

### 2.1. Design of the Randomised Controlled Intervention Study

The recruiting for the randomised controlled study was performed by poster, via the Charité intranet and via the intranet of the Berlin job centre. Participants aged between 27 and 50 years without physical or mental disease were included. Twice a week for seven weeks, the participants took part in either an hour of eurythmy therapy (EYT) under the instruction of a eurythmy therapist with homework (53 participants) or an hour of step aerobics (STA, 53 participants). A further group (15 participants) had anthroposophical art therapy. The emphasis of the eurythmy therapy was on experiencing the connection between physical sensation (of the feet, the spine, etc.), perception of the movement, and experiencing of one's own personal intentionality in the exercises. The exercises included use of the sound “M.” In the M exercise, the hands are held at about the level of the chest with the palms facing each other and encounter each other alternately. This leads to the simultaneous experience of “pushing something away” and “taking something back.” This movement is intended to produce attentiveness to the outside and to the inside at the same time and permit a weighing up between the demands from without and the inner needs. Through the reflecting practice of this gesture, it is possible, for example, to uncover one's own ability, unconscious in daily life, to burden oneself with (perceived) demands or toward them off, and to interpret something as a stressor or not.

### 2.2. Methodology of the Qualitative Study

In addition to the intervention, the subjects were asked to participate in a guided narrative interview after completion of the intervention. The development of the interview guide, the conduct of the interviews, and the transcription of the interviews were performed by the psychologist JK, the eurythmy therapist KP, and a student assistant. The qualitative study was designed after conducting the interviews and uses questions 1, 2, and 5–8 of the questionnaire to answer the question formulated above (Box 1). For this, the verbatim transcriptions of the interviews were analysed by two external qualitative researchers using a hermeneutic content analysis approach (BB) and a body phenomenology (MB) approach. First, all answers were removed from the person-related context and sorted by question. The analyses were performed by BB and MB separately and then compared, discussed, and merged in a complementary fashion. 

*Box 1: (Interview Guide)*

(Questions which were included in the analysis)

(FQ = Further questions, only to be asked if no answers come spontaneously)

*Questions*

(1) What did you expect personally from participating in this study?

 FQ: What positive/negative expectations did you have of the intervention?

(2) How severe was your stress before the start of the intervention?

 FQ: Critical life events?
 FQ: Would you have tried alternative ways of coping with the stress?

(5) Did you notice changes in the stress during the intervention?

 FQ: Were you able to cope better with stress?/Did you develop new strategies and techniques? If so, which?

(6) Did you notice behaviour patterns through the intervention that you were not aware of before?

(7) How do you think the changes came about? What do you think is responsible for them?

 FQ: Intervention/through taking part in a study (being looked after, attention)/sport, exercise, calm, relaxation, group, feeling of responsibility towards the study team, personal motivation, meditation/therapist/relationship to the therapist/important events during the time of the interventions

(8) What sticks particularly in your mind about the interventions? What did you take away from the intervention for the future?

### 2.3. Ethical Aspects

The performance of the randomised controlled study including the qualitative interviews was part of the application for ethical approval submitted to the ethics committee of the Berlin Charité. A positive ethics committee opinion was obtained. Participation in the interviews was voluntary. The study participants signed informed consent confirming that they agreed to the pseudonymised use of the data and that they were aware that they could revoke their consent to participate in the study at any time without giving reasons.

### 2.4. Hermeneutic Content Analysis of the Data

In the hermeneutic content analysis of the data, after the open and axial coding (cf. [6–8]), the codes were grouped, paraphrased (Table 1 and Box 1), reduced, and then interpreted with regard to the research question. The aim was to identify objective complexes of meaning on the basis of the available text. The interpretations made on the basis of these steps were reported as results in continuous text, while the underlying paraphrasing and quotations are listed in table form in the appendix. Qualitative content analysis is a process of understanding complex layers of meaning in the material. The analysis does not stop at manifesting superficial content but is aimed towards latent complexes of meaning [6]. Various philosophers have pointed out the importance of assignment of meaning in terms of a subjectively relevant action. For example, the sociologists of knowledge Berger and Luckmann [9] developed a model of the everyday world in which the acting subjects assign meaning to their experiences and develop interpretations and an underlying logic to their actions which are suitable for everyday use. These acts in turn generate certain effects. This means that the body of daily knowledge of the study participants and their assignments of meaning to the effect of the respective interventions on their stress perception are of therapeutic relevance. In the sociology of knowledge, the concrete assignment of meaning is regarded as fundamental for the societal and subjective reality and hence for the both societally relevant knowledge and subjectively relevant knowledge, feeling, and acting. Thus, from the sociological perspective, the assignment of meaning of the study participants is seen to be important in its own right for the effectiveness of treatments in addition to the respective physiological dimensions studied.

### 2.5. Body Phenomenological Analysis

The body phenomenological analysis, like the hermeneutic analysis, was performed via open an axial coding. Against the background of phenomenology, the primary scientific perspective of this analysis is that of an ecological theory of the organism [10, 11] which interacts complementarily with its environment in the sense of the gestalt circle [12]. The focus is on the response which the eurythmy triggers in the study participant, on the interactions between body, consciousness, and environment. In body phenomenology, the felt body (German: Leib) is the active perceptual agency which precedes the reflective perceptions. It is not the intellect but the felt body which is the medium of experiencing the world [13].

The felt body is also not the “material body out there,” from which a person might be able to distance himself, but the entity with which he is existentially connected and which allows him to be in the world as a perceiving and acting agent. Husserl called this active entity the “functioning body” [14]. Complementary therapies address the senses in numerous ways. The resulting perceptions are the corporeally engaged implementation of the environment as the expression of a person's individual corporeal capacity. The felt body is thus a kind of resonator, actively reflecting his environment [15]. The felt body, which on one hand is coupled with nature and on the other hand belongs entirely to the individual person, is not an entity which can be separated from the material body (German: Körper). The felt body and the material body are, rather, two complementary forms of existence of one and the same entity. For everything which is experienced perceptually via the felt body and in synchronicity with the (therapeutic) environment, there is a correlation in the material body. This explains the effectiveness of all mind-body techniques: perceptions, for example, mindfulness, meditation, artistic exercises, and correlation with the processes in the material body, for example, improvement of the immune status and lowering of blood pressure [16, 17]. The phenomenological approach searches for signs of such felt-body processes and thus distinguishes in the data analysis between

1. a priori experience: indicators of immediate experiencing of intentionally perceptive processes before any judgment;
2. reflected experience: indicators of experiences that have already been thought through.

The codes stem mainly from the verbatim responses of the subjects. In some cases, they are paraphrases for the purpose of summarizing longer responses. All codes were incorporated in the continuous text.

## 3. Results

Of the 106 study participants, 76 participated voluntarily in the qualitative interviews. Of the 76 interviewees, 36 were from the eurythmy therapy group (f = 31/m = 5) and 40 were from the step aerobics group (f = 28/m = 12).

### 3.1. Hermeneutic Content Analysis (BB) 

#### 3.1.1. Expectations of Participation in the Study (Question 1)

The participants took part in the study with various expectations. Some had no expectations, others had the diffuse expectation that they would be given something that would help them cope with stress. Others again expected that they would be introduced to a new form of exercise and a fourth group was interested mainly in getting to know complementary methods.

*Getting to Know New Methods for Stress Reduction*. Many participants wanted to learn about ways of coping with stress. As a rule it was not clear what individual participants understood by stress. Little was said about their current life situation. Some participants used formulations such as “stress reduction, winding down, finding peace and quiet, and being able to relax.” It is possible that these participants had an underlying stress model which assumes an excess of external stressors (at work, in the family). Others used expressions such as “wanting to take a step back, not letting things affect me so much anymore, and not letting things get to me so much”. Participants occasionally said they wanted to “learn more about themselves.” This can be assumed to indicate a willingness and interest in personal further development. These participants seem to have a different underlying stress model in which the responsibility for coping is seen to lie in the individual intrapsychic situation and capability for appropriate appraisal. Participants also expressed quite concrete ideas about what they wanted to deal with, for example, sleep problems or feelings of depression.

*Being Introduced to New Forms of Exercise*. Another expectation of the study was the possibility of access to new also previously unknown forms of exercise. Some of these were participants who were unable to carry on with a previously practiced sport and were looking for a substitute. Others wanted to stop being a couch potato and get moving again.

*Interest in Taking Part in a CAM Trial.* Interest in complementary medicine and the opportunity to take part in a trial and thus learn more about a certain intervention characterised the motivation of a further group of participants. Other patricipants motivation was the opportunity to support CAM—treatment options by taking part in such a study. An other factor that might play a role here is that it possibly seems easier to try out an unknown treatment method in the context of a study and its controlled conditions.

#### 3.1.2. Stress Level before the Start of the Intervention (Question 2)

The participants reported different levels of stress before the beginning of the study. However, it remains unclear what the interviewees understood by stress and how it was defined. A few interviewees mention this as a problem in their response. Amongst those who rated their stress levels as high, the following stressors could be identified: stress through (1) multiple pressures and demands in different combinations, (2) work-related pressures and demands, (3) internal beliefs and attitudes, or (4) family pressures (see Table 1).

#### 3.1.3. Changes as a Result of the Interventions

For this analysis, questions 5, 6, 7, and 8 were analysed together (See Box 1).

*(i) Results of the Eurythmy Group.* The following categories were identified.

*(1) Integration of Mind and Body through the Combination of Speech, Breathing, and Movement*. The combination of these three elements creates a movement which can be reproduced mentally or internalized. The body responds to stress situations with physical symptoms. Through the exercises, it becomes possible to experience and recognize these reaction patterns and thus to link them to events in the environment and reflect on them.

*(2) Creation of a Personal Inner Space.* The metaphors of the empty room and the inner space describe the fact that these are evidently previously unknown perceptions which are unaccustomed and new and, like an empty room, ask to be filled. Something can enter this space for which there was evidently hitherto no (perceptual) space, something which can come between an external event and the internal reaction.

*(3) Perception of the Felt Body through Expansion of the Material Body by Its Subjective Dimension*. Through the newly acquired perceptual space, an appraisal agency can develop which is able to create distance, pause for a moment, and consciously draw a boundary.

*(4) Achieving a New Balance between the Internal and External Worlds*. The previous boundaries between the internal and external worlds can be redefined. Where previously external events crossed one's personal boundaries, these boundaries can now be redrawn. The stress-precipitating factors can be left outside and do not invade the inner world.

*(5) Increased Options for Action*. The improved perception of one's own body and one's personal needs creates space which can be made use of by a self-aware subject. This feeling of empowerment makes it possible to cast off the feeling of impotence. New choices become visible. It is possible to put the brake on, slow down or speed up, and to decide for oneself. 

*(ii) Results of the Step Aerobics Group*

*(1) More Zest for Life, Tranquility, and Relaxation*. Some participants were able to enjoy the exercise. It distracted them from their everyday problems and enabled them to relax and sleep better. Performing exercises in a group was experienced as pleasurable. Their motivation to participate in sports was stimulated.

*(2) More Relaxation through Concentration.* The need to concentrate on the exercises helped some participants to relax better.

*(3) Benefit: No Substantial Change in the Stress Symptoms*. With regard to the stress perceptions, there was little mention of change. The pressures and demands experienced as stress had not changed. Some participants even complained of additional pressure, particularly if the surroundings did not seem quite right (see Box 1).

### 3.2. Body Phenomenological Analysis

#### 3.2.1. Results in the Eurythmy Therapy Group

The a priori experience of the participants is expressed by the category classes,* sensation* and* perception*. Experiences already reflected on could be divided into the classes* reflection* and* options for action* and* what remains*.

*Sensations and Perceptions a Priori*

*Feeling Moved*. This category describes a group of characteristics which indicate an awareness of something that cannot yet be expressed in clear ideas or words. In perceptual psychology, these are sensations [18]. This includes statements such as “sort of reached me” (m22876a3), “felt in good hands” (w24668a3), “felt kind of pleasant” (m15281a1), and “the rest phase really feels good” (w12471a2), but individual participants also said that “all this waving your arms around” (w03681a1) “felt silly” (w03681a1).

*Walking into an Empty Room*. By far, the majority of the statements on the effect of the eurythmy therapy describe concrete perceptions or mental concepts. In contrast to pure sensation, perception describes the result of a complex process by which “events and objects are ‘rendered experienceable'”. Perceptions have direction and weight, are spatial, have gestalt character, categorize the perceptual content, and can be recalled as mental concept. The ambivalent experience of experiencing “concentration” (w03064a1; w14568a3) and “relaxation” (w11864a2) is expressed in the movement. What happens is a “switching off and concentrating entirely on the exercise” (m00980a1). Thus, it does not appear to be a contradiction that during an experience characterised as “totally relaxed – absolutely” at the same time a “sharpened sensory perception” (w19561a3) develops.

An important experience is “experiencing structure” (w14568a3; w10366a2), first in the movement sequences. “Boundaries” (w10366a2) are experienced. This also creates a kind of body memory: “I feel as though it's got inside me and I can take it with me now” (w24783a3) “When you do (the exercises) often you feel a kind of resonance.” (w09280a1) This feeling of resonance can, if needed, also be generated from memory and without the eurythmy exercise: “…that it is very helpful in certain (stress) situations to create visual images which you can then use” (w14672a3). In stress situations, this makes it possible “to set boundaries, for example” (w14672a3).

“It is like a kind of – I'm exaggerating now – ‘other world' which you enter” (m22876a3). Some subjects feel they lack “the proper words” (m22876a3) for this perception. The common feature is the experience that through the eurythmy therapy the participants have a tool which they can use to create their own conscious space. “This resulted in a feeling of calm, a calm anchor, everything was calm” (m22876a3). “…well the exercises have – how can I put it – stayed in my body; if you do them often then they're always there with you” (w09280a1). The mental image and the movement go hand in hand and the mental image is enough to conjure up the physical experience of a calm space: “…well you arrive there and you're in a completely different situation, regardless of where you were before, it's as though you walk into an empty room and then first empty yourself completely too, free yourself of everything” (w14672a3).

*Reflections*

*Shows You Where You Stand.* Typical statements are “winding down [and] staying centred” (w04862a1) and “learning a lot about yourself” (w12661a2) and eurythmy therapy is something that “shows you clearly where you stand at the moment” (w14672a3). It allows you to “connect with yourself. I mean through the movements and the speech and the harmony of speech, movement and sensation” (w20773a3). However, “it's inexplicable to me how it works” (w20773a3). This taking a step back gives you time to reflect: “Hey, what other options are there out there so I do not act as though I'm blinkered and get all het up but try and find a different way of seeing things” (w24783a3). Unproductive behaviour patterns become conscious and can be worked on. Awareness of oneself and one's environment is heightened. What remains is “just allowing yourself to pause for a moment and look at exactly what's going on” (w24783a3).

*Options for Action*

*Being Able to Leave the Stress Outside*. You now have the option to “take a break” (m00980a1) or to not “let things get to you so much” (w10366a2). You can consciously “leave [a stressor] outside” (w10366a2) or “keep the level below this tip-over point or before this tip-over point and could open the valve first and release the pressure” (17669a3). Exercises can be done at home or at work and “I can also do the exercises in my mind afterwards if I do not have the opportunity to do them at that particular moment” (w11864a2). Eurythmy therapy is an individual tool against stress, can be incorporated in your everyday routine, and help you to “downshift” (w17669a3). Eurythmy therapy allows you to experience “But this is my time for myself, that I can always reconnect with myself” (w17074a3) and just “get some distance and simply say with some things I really do not have to do so much” (w06779a1). 

*What Remains*

*Inner Calm*. “I cannot prove it but I feel calmer, more composed, inwardly stronger” (w04862a1). The eurythmy therapy has created “inner composure” (w09280a1) or “grounded [me]” (w10366a2). It has “[made me] totally relaxed” (w19561a3) and feel “unbelievably good” (w12471a2). In addition, it led to a general sharpening of attention (w10177a2).

#### 3.2.2. Results of the Step Aerobics Group

The data set generated by open coding could be divided into the classes* Physical, Emotional Experiences and Perceptions* and Evaluation of the Benefit.

*Physical, Emotional Experiences and Perceptions a Priori.* The sensations and emotional experiences are summarised here to begin with describing experiences of consciousness which, in contrast to perceptions, are prereflexive and cannot be clearly classified [18].

*Physical Wellbeing*. The exercises were experienced as physically pleasant (w23065b3). “Well more a sense of physical well-being, but that your head is cleared of the problems and thoughts: no” (m05582b1). The experience is that “you feel good during the exercise and also for a short time afterwards” (w19477b3).

*A Pleasant Diversion*. The main element is enjoyment: Taking part in the step aerobics “was amusing” (w01769b1), “easygoing” (m20585b3), “nice” (w01769b1), and “was fun” (w09771b2). Apart from these more emotional experiences the participants less often mentioned the influence that the STA had on their physical sensation: “exercising to music was pleasant” (w14380b2); “exercise makes you feel good” (w09771b2). There was practically no mention of a connection with the experienced stress. A typical statement was “was a pleasant diversion” (w03179b1).

The following statements were reflected cognitions of the participants about their experience. 

*Sharpened the Senses/Had to Concentrate Damned Hard*. In the words of one participant, it was like “having a mirror held up in front of things again, where you could look at them cognitively, so that you get calmer and can change your lifestyle” (m12182b2). The following statement exemplifies how the STA forced the participants to focus their attention: “you had to sharpen your senses, to so-to-speak give the body the commands which were expected. And because you were in a group you also made an effort to be good and not get out of line. Now you forgot everything going on around you, I mean stress from work and things like that” (m16777b2). The result is a time-out, which can however also create stress itself “if I'm only desperately trying to keep up and looking to see which movement comes next” (w03574b1).

A not insignificant number of participants felt that one stress was replaced by another: “well one thing is the physical side and the other, and I think that's more what causes the stress that you feel, is the head, and it's really hard to believe how challenging it can be for the brain to remember a simple sequence of movements and carry them out at the same time and that's so complicated for my brain” (w23483b3). The participants accepted the fact that “you had to concentrate so damned hard” (w14380b2). But for some, the rapid sequence of exercises was “too short to achieve any kind of relaxation” (w03574b1).

*Evaluation of the Benefit*

*Start the Evening Relaxed after the Exercise.* As a result of the STA, some participants were able to “really switch off after work” (w03471b1). In many statements, the focus was on the experience of the STA as sport and this was generally evaluated as positive: “sport was good for my muscles” (w13661b2) “… for the immune system” (w23483b3), and for the “cervical spine” (m16777b2); “Even though it was physically strenuous I still went home feeling fit. I definitely leave with a positive result” (w14380b2); there seems no doubt “that exercise is good” (w03471b1).

A number of participants reported that after the STA they were able to “start the evening more relaxed” (w12764b3). However, there were also statements such as: “Well at the moment I cannot say that it's brought about any lasting change. On the day itself yes” (m24376b3). The relaxation appears to be directly connected with the physical participation in the exercises. A lasting effect is generated at best through the motivation to take up sport again or to do sport more often. For example, one participant reported “well actually no substantial change, I mean that I did not really feel less or more stressed. What it did do for me was that I felt more motivated to do sport” (w09771b2). This experience of a call to action was felt by numerous participants: “…that maybe I should take up sport, something, I do not know what, but I should look for something” (w14380b2). It stands out that the basic attitude to STA as a sport is an affirmative one: STA is regarded as positive not only because of the positive subjective experience but also because of the society that generally regards sport as positive or at least beneficial for health.

*Temporarily Clears Your Mind.* Some participants explicitly commented on the effect of the STA. One sums it up like this: “Yes one thing is the physical side, and the other really what goes on in your head, that at the moment I have no room for anything else, although otherwise so much fits in there, yes and I think that's the main thing, the main reason why you can switch off so well, probably with any kind of sport too, it could probably just as well be something else instead of doing things on the stepper” (w23483b3). The effect is “a clear cut the moment you enter the room” (m24376b3). “Well while I was really coming here twice a week I had the feeling that lots of things just rolled off me like water off a duck's back” (w01769b1).

Various participants explicitly said that they did not notice any change. One example: “I work out regularly anyway, so I cannot say that there was any stress reduction” (m26061b3).

### 3.3. Comparative Evaluation of the Results

If we place the results of the different analytical approaches side by side, we can see that they complement each other. The body phenomenological analysis keeps strictly to the perceptions of the study participants. The hermeneutic interpretation applies itself to the hypothetical disclosure of latent contextual associations.

Thus, while the body phenomenological analysis shows the response of the body to a therapeutic intervention, the hermeneutic interpretation points to the meaning that this response phenomenon has for the individual. Both analyses reveal the presence of a previously nonexistent perceptual space. The following interpretations of the combination of both main analytical categories emerge.

#### 3.3.1. For the Eurythmy Therapy Group

*(1) Enabling a Productive Therapeutic Response*. Therapeutic interventions create an environment with which the intentional consciousness can synchronize (with regard to the threshold experience, e.g.). In particular, the eurythmy therapy practiced here permits productive perceptions (perceptions, feelings).

*(2) Emergence of a New Perceptual Space.* These perceptions include the experiencing of a new “perceptual space.” This can be charged individually with sense and meaning and creates the awareness of a manageable entity (intentionality). 

*(3) Reevaluation of the Accustomed Perception.* This entity is able to reflect on and reevaluate the previously unreflected, habitualised patterns of action. This reevaluation can apply to both the externally perceived stressors and the inner resources.

*(4) Emergence of New Options for Action.* On the basis of this reevaluation new options for action are perceived and can be actively utilized.

#### 3.3.2. For the Step Aerobics Group

The step aerobics places increased physical and intellectual demands on the subjects. These are perceived by them in different ways:

1. pleasant and relaxing;
2. insufficiently challenging and/or boring;
3. too challenging and thus experienced as stress-enhancing.

*(5) Descriptive Statistics of the Responses in the Qualitative Interviews with regard to the Changes in Stress (Response to Question 5)*.* Did you perceive changes during the intervention with regard to the stress? *(See Table 2).

## 4. Discussion

The qualitative interviews show that the participants did perceive effects of the interventions. Both the step aerobics group and the eurythmy therapy group reported feeling relaxed as a result of the intervention. However, it stands out that practically only the participants in the eurythmy therapy group achieved a changed perception of the factors which they had previously experienced as the causes of their stress. The results of the analysis of the STA group do not point in this direction; hence, the exercise alone cannot be regarded as an adequate intervention for stress reduction. In addition, the positive affirmative attitude to sport in society must also be taken into account when evaluating the results.

### 4.1. Stress Model

In stress research, Kaluza [19] distinguishes different stress models. The medical epidemiological model focuses on quantity and quality of stressors. In the psychological stress model of Lazarus, it is the subject that is responsible for whether an external stressor is regarded as a challenge or a burden and that also evaluates their own potentials for dealing with the challenges or pressures as adequate or inadequate [20]. The salutogenic stress models look at the individual's own resources and the perceptual possibilities [19].

The interviews with the participants show that they evidently had different inherent stress models. While some interpreted stress as too much of something (work, customer contact, engagements, shifts, further training, family, etc.) and thus used an epidemiological stress model, others focused more on their attitudes and their reaction to the stressors. They saw an inability on their part to set boundaries or a self-imposed pressure to do everything perfectly. A third group saw themselves as lacking inner resources, as problems appeared insolvable and they were unable to continue their development. The underlying stress model here may be a subjective stress model which assumes a lack of salutogenic resources.

If we now look at the results with regard to the different stress models held by the study participants, we can postulate that the different models have an influence on whether an intervention can be interpreted as stress-reducing or not. It is striking that it was mainly those who had experienced a change in their own perception reported a subjective effectiveness of the interventions: these were almost exclusively participants from the eurythmy therapy group. According to their statements, the eurythmy therapy can create an independent perceptual space, which opens up internal possibilities to actively distance oneself from external stressors. This consciousness space appears to be an important precondition for reevaluation of both external and internal stressors and increased perception of resources. The inner distance, the deceleration of their own actions, and the taking a step back create the necessary space for a subjective reevaluation. However, the emergence of this inner space was only made possible by the exercises in the eurythmy therapy group.

Eurythmy therapy can be classed as a mind-body technique and requires the active involvement of the participant. Performing the exercises, formation of mental images, and experiencing the movement, feelings, volitional impulses, and other perceptions are not passively experienced but actively generated and thus generate an awareness of one's personal perceptual space. This active generation of perceptions presumably correlates with therapeutic effects. An important aspect of participation is the respective stress model held by the person performing the exercises. Participants who have an externalized stress model and feel at the mercy of their stress appear to have few possibilities of changing their perception and their coping strategies in this productive way. The study results suggest that it was particularly those participants who had or developed a stress model which resulted in re-evaluation of the stressor and of their personal resources who benefited from participation in the study. This productive approach to the treatment in this study was seen almost only in the eurythmy therapy group. The self-perception created through the eurythmy therapy permits a re-evaluation of external stressors and inner resources.

### 4.2. Gender Differences

With 59 female and only 17 male participants, we find a common pattern of gender allocation within this study. In a recent study about the use of CAM methods in Norway, which find out, that a total of 33% of the participants reported use of CAM within the last 12 months, 42% of the asked women used CAM methods compared to men (24%). As reason for this imbalance, the authors cited qualitative studies, where women express unmet needs regarding their individual health care goals. They emphasize the importance of CAM as a health care system that enables them to take active part in decision-making processes and treatment and, thereby, contribute to positive health outcomes for themselves as an important basis for their treatment decisions [21]. Because participants have been allocated by randomisation, the higher amount of women mirrors only their higher interest in CAM methods compared to men, regardless of the allocation to the control group.

A limitation of this study is that the inclusion criterion for the healthy participants were raised stress scores in the TICS questionnaire (average *T*-score in the eurythmy therapy group: 60 and that in the step aerobics group: 59). It must be assumed that these participants are able to mobilize further resistance resources which were not documented here. A further limitation is that the decision to include qualitative interpreters in the study was not made until after the data collection so that we had to deviate from the usual rules of interview guide compilation and sampling strategy. The questions of the interview guide could only partly be analysed. The results reported here are based on the analysis of the qualitative statements.

## 5. Conclusion 

The qualitative results provided revealing insights into the profound effects and subjective assignments of meaning to external and internal stress factors. Processes of mental reinterpretation leading to stress reduction can be stimulated by physical procedures such as eurythmy therapy.

## Figures and Tables

**Table 1:** 

Categories for level of stress before the study (Question 2)	

(1) Excessive stressors	
(i) Combination of numerous demands and pressures through career, looking after children, household, professional development courses, freelance work, or home construction. (ii) Having to manage too many projects at the same time (too many irons in the fire). (iii) No longer able to cope with the pressure.	“*Yes, a kind of subjective feeling of not being able to cope with all the demands at the same time, in my private life and in my job, although I can't say that there's any concrete pressure from anywhere that I could actually name, it's more just everything together, the fullness of the day you might say.” (m22876a3)* *“I was totally tense and almost dreaded every new task I had to deal with, whether it was just a simple everyday matter or a new project at work, because I notice that I can't stand any more pressure, and I felt totally stressed, well actually almost on the brink.” (w14672a3)* *“Always stress. But then maybe I also create it myself, hectic, always on the go but not so that I would say “I'm stressed out” and burst into tears, it's not like that. I think I need it too*” (m08563b2)

(2) Work-related stressors	
(i) Too long working hours (two jobs) or shifts that go against the body's natural rhythms. (ii) High (physical) demands (noise at school; high level of customer contact). (iii) Uncertainty through time-limited employment. (iv) Various work projects with colliding time schedules. (v) Taking on responsibility as team leader. (vi) Difficulty getting on with colleagues (dominant colleagues; relationship problems with colleagues).	“*It's an ongoing problem with me, stress I suppose, also sort of a kind of eustress, I'm not a very calm sort of person, always in action and yes, I try to learn to handle it, handle it better, so that I don't end up with exhaustion, which has often happened in the past.” (w03471b1)* *“Actually it's usually rather high, through the customer meetings which then often don't go in the right direction and then of course I don't get anywhere with the customer. Yes, it's the work is very nice but it does involve very strenuous meetings,…” (w23065b3)* *“Well I did have quite a strong feeling of stress, all of a sudden I had a study that I had to analyse, with 5000 variables and then I also had another study for which I am actually employed and have a contract, but because of this other study I had to as it were postpone the work, and that led to stress.” (m12182b2)* *“Well the problem is that there are just too many things in my life that are important to me and so …so I find it difficult to put the focus on any one thing. Well, so there's uni, 2 children, working 2 days, although I find the work incredibly fulfilling. (…) I don't really experience it as stress because it gives me a lot and well it's a vocation really. But then altogether it is stress because I can't do things which I could be doing during that time. (…) But there are only 24 hours in a day. My flat gets more and more chaotic and actually I spend a lot of time getting things sorted “inside me”. Actually, I could say that I haven't got the time to study and work*.” (w06375a1)

(3) Own beliefs and attitudes	
(i) Feeling of being under too much pressure. (ii) Inability to recognize one's own limits early enough. (iii) Feeling unable to cope with the demands. (iv) Desire to do everything perfectly. (v) Attribution of the excessive pressure to the amount of work. (vi) Feeling of not wanting to miss anything.	*“Well, that varies, I mean there are days when I can cope with it well, with stress, but sometimes I also notice that I get into a sort of pattern, that I find it difficult to distance myself from it, that then in a job one job has to be done after another without stopping for breath, then I notice that I get caught up in it too, well yes I think it is a high level of stress.” (w03064a1)* *“I felt relatively stressed, although I, I mean I really should say that I always create the stress myself. I mean I haven't really got that much stress at work, that I always run around or have a lot of stress privately, ok my mother's death maybe, I create a lot of stress for myself too, that I think everything has to be perfect.” (w04108a1)* *“Significant, or substantial. Well I did feel rushed, from one appointment to the next, things like that, so that sometimes I just didn't have the time to calm down, although I had already done 2 or 3 relaxation techniques before”. (w05367a2) *

(4) Family conflicts	
Insoluble family problems which dominate one's whole life are perceived as stress; this includes seemingly insoluble problems with one's own parents or problems which have been pushed aside too long. Being prevented from continuing on one's own path can be perceived as stress.	“*And then a relatively large amount of stress privately too, when there were sometimes these unsolvable conflicts, when I noticed that that throws me off course, that it's too much.” *(w06779a1)

Changes in perception in the eurythmy group	

(1) Integration of mind and body through the combination of speech, breathing, and movement	
The combination of speech and movement enabled the participants to create a connection between their cognitive attitude and their bodily perception. Through visualisation these body images can be summoned up again and lead to changed behaviour in everyday situations.	*“I find this combination of movement and speech much better than if you only have one or the other.” (w24264a3)* *“…because it's also easy to incorporate movements in your everyday life and even if it's not the movements, then the sentences which were spoken there, and that they just stay inside you and that you can make that clear to yourself again and again, that there's a different way of doing things and that you just have to become aware of it when you're racing along in the fast lane like that, and that you can also reduce speed again now and again.”* (w17669a3)
Reducing speed in everyday life expands the space for one's own perception. Breathing can be perceived as a relevant mediator. Participants usually notice that their breathing is too shallow; deeper breathing is perceived as helpful and brings relief and permits calmness.	“*Yes, I've noticed a behaviour pattern, that I do everything relatively quickly, that's my behaviour pattern, I've become aware of that, I didn't notice that before*.” (w03064a1) “*I learned from the therapy to really consciously breathe now and again, breathe in and out, that life is not so hard after all. I mean this listening to yourself and then you can wind down better and you're calmer somehow…” *

Creating a personal (inner) space	
Perception of the felt body though expansion of the material body by its subjective dimension	
The exercises make it possible to intensify the perception of the body and permit a greater consciousness of physical processes and thus of the stress symptoms. Recognizing how difficult it can be to get away from (negative) emotions. The exercises provide the participants with a tool which makes it is easier to distance themselves inwardly from things that usually irritate them. The improved self-perception makes it possible to take a step back and look at the situation, creates space for appropriate appraisal, and extends the options for action. The inner appraisal increases tolerance towards others.	“*Through the movements I can intensify my sensations and thus increase my awareness for myself and my body and my state. When I notice that I'm losing my grip on something, I'm going to try and remember that” (w20773a3)* *“The exercises have already sort of stayed in my body. If you do them often then it's sort of always there with you and I think once, I wasn't feeling so good, then I did the exercise in my mind while I was cycling and then I felt better… and if you do that often then your body remembers.” (w17074a3)* *“…not letting other people get so close to you so that you don't get emotional” (w09280a1). “There are exercises that I have in my head” (w01166a1)* *“Well, behaviour patterns, that I find it hard to let go of my anger, that there probably must be some sort of incentive, like these exercises, I mean that I have to decide consciously to do an exercise like that, now I'll let go of that, and afterwards you have a good feeling which you wouldn't have had if you had just let things go on.” (w01061a1)* *“…more an awareness that I'm so fidgety and do not radiate calm, I'd like to change that” (w24164a3)* *“Combination of rest and movement, finding balance between rest and movement, tension and relaxation, being able to direct your thoughts inwards and outwards, being mindful of yourself and being mindful of others, and finding the right balance between the two.” (w04180a1). “The ability to feel exactly where I stand at a particular moment: at rest or in action, and then also to consciously deal with it and be able to shape it” (w24964a3) *

Achieving a new balance between the internal and external worlds	
The more conscious awareness of one's own body makes it possible to distinguish more consciously between self and other and draw boundaries between the two. One is no longer so other-directed. Doing the exercises in a group allows the participants to recognize their own behaviour or see it mirrored.	*“That you can leave others their rhythm and still stay with yourself and be aware of your own rhythm” (w10177a2)* *“…the coming and going of the rhythm of life, breathing in, breathing out, letting in and letting go, not holding on to things” (w10366a2)* *“Yes, I find it very exciting, when you're together in a group of people who all come with the same symptoms, I mean stress, (…) but in watching the group I recognized things in myself and that was exciting, to see it from the outside like that, that really helped me, I must say.” (w24264a3) *

Perceiving new options for action	
The ability to break out of the victim role and assume responsibility for one's own perceptions and reactions. This ability leads to the perception of new options for action and thus generates a freedom of choice of which the participants were not previously aware.	*“Keeping calm in the movement so that I still have the freedom of choice even when there's a lot going on, not becoming a victim of the circumstances and the demands but assuming responsibility for the situation myself at every moment.” (w24783a3) *

Perception of changes in the step aerobics group	

More zest for life, tranquility, and relaxation	
Movement helps to wind down when you are angry. Movement relaxes and creates distance; you can see things more calmly.	“*Well I'm more laid back about things, I can definitely say that, if I can't change things I just have to accept them, there's a solution for everything.” (w02766b1)* *“Relaxed afterwards, I mean not tired, that you felt emotionally strengthened again, went home in good spirits and your head was clear.” (w03471b1*)
Concentration improves the ability to relax	“*I wasn't aware that I couldn't concentrate properly at all, but that that is important so as to be able to switch off properly again and relax” (w16263b2*)
Participating in the study increased the motivation to take up sport again. The active exercises made it possible to regain a feeling of lightness and carefreeness and thus had a beneficial effect, in contrast to passive wellness activities such as massage. The sport made the participants feel good and was experienced as fun and was also experienced as relaxing in the group.	*“…sport is an exaggeration, but getting a bit of exercise and stretching and things like that, and I've taken a bit of that home with me” (w22059b3)* *“…yes, it was simply a good feeling because it was also fun. If it didn't go so well at the beginning and at the end it did, then it was worth the effort.” (w25467b3)* *“Yes, exactly, I was sort of in my own world during these exercises. I'm amiable, I mean I remembered my childhood, when you sort of skipped around without a care and simply enjoyed life.” (m23065b3) *

No fundamental change in the perception of stress	
The intervention did not bring about any fundamental changes in the stress perception. During the exercises you can feel good and relax but they do not bring any lasting relief. The problems are still there.	*“Well more a sense of physical well-being, but that your head is cleared of the problems and thoughts: no” (m05582b1) *
There was even one participant who experienced doing the exercises as an additional stress factor. Participants who do sport regularly experienced no reduction in stress.	*“No, not really. Well the sport business was always very relaxing, but generally now, work still stays in the way as an obstacle.” (m05976b1)* *“Not at all, I mean nothing positive. It's just as stressful as before at work.” (m26061b2)* *“Well anything that looks like step aerobics (…) is horror for me” (w166161b2). *
The group situation leads to a certain feeling of being under observation. However, the pleasant group atmosphere in which no-one is judged is perceived as positive; however, participants denied any change with regard to the perception of stress.	*“Not really. I mean in the group there is the group effect too, whether you're keeping up or not, or whether you're are sort doing it a lot worse than the others or whether that's ok, that's always the thing in a group, (...). The atmosphere was so friendly. You didn't have the feeling that you might be a failure or something like that, no.” (w23761b1) *

**Table 2:** 

*N* = 76	Eurythmy therapy group		Step aerobics	
	*n* = 36		*n* = 40	
	Female	Male	Female	Male
	*n* = 31	*n* = 5	*N* = 28	*N* = 12
No (*n* = 26)	1	0	16	9
No lasting change (*n* = 11)	3	3	3	2
Yes (*n* = 39)	27	2	9	1
